# Interplay of protection and damage through intermolecular processes in the decay of electronic core holes in microsolvated organic molecules†

**DOI:** 10.1039/d4cp03907f

**Published:** 2025-03-06

**Authors:** Dana Bloß, Nikolai V. Kryzhevoi, Jonas Maurmann, Philipp Schmidt, André Knie, Johannes H. Viehmann, Catmarna Küstner-Wetekam, Sascha Deinert, Gregor Hartmann, Florian Trinter, Lorenz S. Cederbaum, Arno Ehresmann, Alexander I. Kuleff, Andreas Hans

**Affiliations:** a Institute of Physics and Center for Interdisciplinary Nanostructure Science and Technology (CINSaT), University of Kassel Heinrich-Plett-Straße 40 34132 Kassel Germany dana.bloss@uni-kassel.de hans@physik.uni-kassel.de; b Theoretical Chemistry, Institute for Physical Chemistry, Heidelberg University Im Neuenheimer Feld 229 69120 Heidelberg Germany; c European XFEL Holzkoppel 4 22869 Schenefeld Germany; d Deutsches Elektronen-Synchrotron (DESY) Notkestraße 85 22607 Hamburg Germany; e Helmholtz-Zentrum Berlin (HZB) Albert-Einstein-Straße 15 12489 Berlin Germany; f Molecular Physics, Fritz-Haber-Institut der Max-Planck-Gesellschaft Faradayweg 4-6 14195 Berlin Germany; g Institut für Kernphysik, Goethe-Universität Frankfurt Max-von-Laue-Straße 1 60438 Frankfurt am Main Germany

## Abstract

Soft X-ray irradiation of molecules causes electronic core-level vacancies through photoelectron emission. In light elements, such as C, N, or O, which are abundant in the biosphere, these vacancies predominantly decay by Auger emission, leading inevitably to dissociative multiply charged states. It was recently demonstrated that an environment can prevent fragmentation of core-level-ionised small organic molecules through immediate non-local decay of the core hole, dissipating charge and energy to the environment. Here, we present an extended photoelectron–photoion–photoion coincidence (PEPIPICO) study of the biorelevant pyrimidine molecule embedded in a water cluster. It is observed and supported by theoretical calculations that the supposed protective effect of the environment is partially reversed if the vacancy is originally located at a water molecule. In this scenario, intermolecular energy or charge transfer from the core-ionised water environment to the pyrimidine molecule leads to ionisation of the latter, however, presumably in non-dissociative cationic states. Our results contribute to a more comprehensive understanding of the complex interplay of protective and harmful effects of an environment in the photochemistry of microsolvated molecules exposed to X-rays.

## Abstract 2

Intermolecular energy and charge transfer from inner-shell-ionised water towards neighboring organic molecules is investigated.

## Graphical abstract

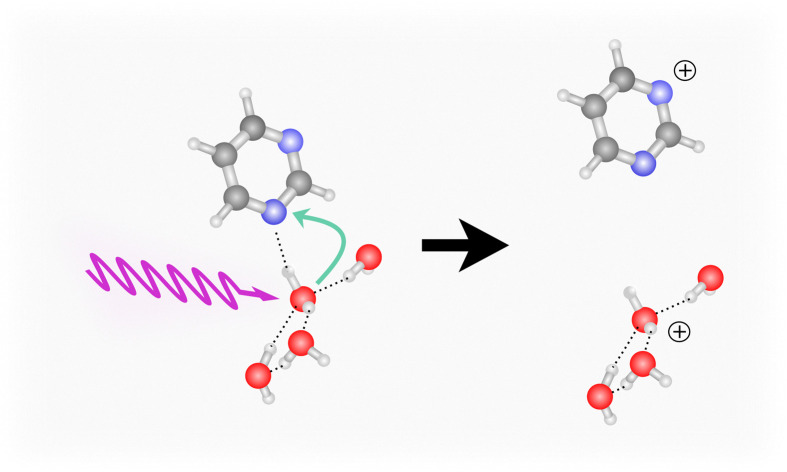

## Introduction

X-ray-induced electron and nuclear dynamics are key topics in research on molecular radiation damage. In particular, understanding the fundamentals of radiation damage to DNA or its constituents is of utmost importance in medicine and biology.^1–8^ The experimental investigation of X-ray-induced processes of DNA building blocks on a molecular level, however, is challenging, if carried out in its natural environment. This environment has a decisive impact on the damage introduced to a biomolecule after interaction. The formation of secondary low-energy electrons,^1,9–13^ radicals,^1,9^ or ions^14–18^ typically are harmful for DNA, whereas the suppression of dissociation processes due to a liquid environment by steric hindrances or neutralisation of charges may protect DNA.^19,20^ In essence, therefore, the net effect of an environment for radiation damage is still not fully clear.

Inner-shell ionisation of an isolated pyrimidine molecule (C_4_H_4_N_2_), for example, initiates Auger decay, leaving the molecule in a doubly positively charged and therefore dissociative state. The result is fragmentation into two singly charged fragments.^21–24^ In contrast, for inner-shell-ionised pyrimidine embedded in a water environment, intermolecular charge- and energy-transfer channels with neighbouring molecules open up which compete with the local Auger decay, preventing the formation of doubly charged states in the molecule, and, therefore, its dissociation due to Coulomb repulsion.^19^ While experimentally the effect has not yet been quantified, theory predicts that already solvation by four water molecules is enough for the intermolecular channels to outpace Auger decay. Importantly, intermolecular decay of the inner-shell vacancy leaves the biomolecule intact in a neutral or singly charged state.^25,26^ The intermolecular decay processes of electronic core-level vacancies considered here are energy- or charge-transfer mechanisms like core-level interatomic/intermolecular Coulombic decay (core-level ICD)^27–29^ and core-level electron-transfer-mediated decay (core-level ETMD).^29^

**Fig. 1:**
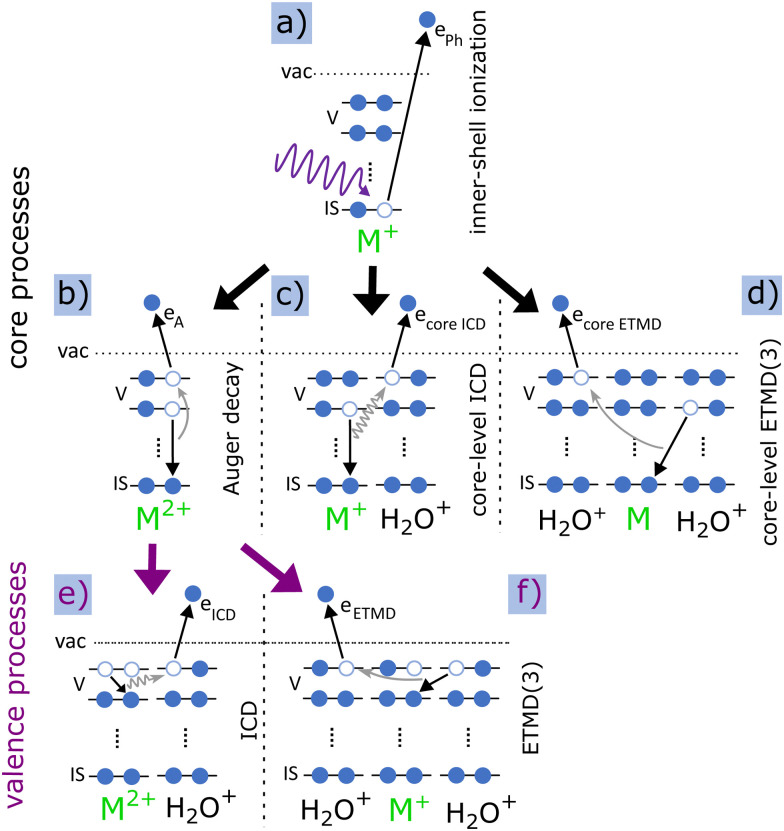
Sketched local and intermolecular processes after inner-shell photoionisation of pyrimidine (M) embedded in a water cluster, grouped in mechanisms involving the core [(a)–(d)] or valence states [(e) and (f)]. Process (a) is the inner-shell (IS) photoionisation of a molecule M emitting a photoelectron *e*_Ph_. Panel (b) displays the subsequent Auger decay where a valence (V) electron fills the core vacancy and another valence electron from the same molecule is emitted (Auger electron *e*_A_). Process (c) shows the core-level ICD, transferring the released energy to a neighbouring water molecule and ionising a valence electron (*e*_coreICD_). Process (d) sketches core-level ETMD(3). Here, a valence electron from neighbouring water fills the inner-shell vacancy of the initially ionised molecule and a valence electron (*e*_coreETMD_) from another water is released. ICD and ETMD can additionally happen after a preceding Auger decay [processes (e) and (f)].

Fig. 1 illustrates the relevant processes, starting with the inner-shell ionisation [panel (a)], which leaves the molecule singly charged and highly excited (M^+^). Subsequently, local Auger decay [Fig. 1(b)] may take place, where the core hole is filled by a valence electron and another valence electron is ejected, leaving the originally singly charged ion in a doubly charged state (M^2+^). Typical lifetimes of these processes are in the femtosecond regime, and, therefore, much faster than any fragmentation dynamics. Competing with Auger decay, the released energy from the filling of the core vacancy can ionise a valence electron of a neighbouring molecule *via* core-level ICD [Fig. 1(c)]. Core-level ICD produces a water cation and leaves both molecules singly charged. Alternatively, core-level ETMD [Fig. 1(d)] can take place. Two variants of ETMD are possible: ETMD(2) or ETMD(3), depending on the number of involved molecules.^30^Fig. 1 only sketches ETMD(3), typically dominating over ETMD(2).^31^ In the former, one water-neighbour valence electron fills the core hole in the initially ionised molecule and the released energy is transferred to yet another water emitting one of its valence electrons. Consequently, ETMD(3) produces two water cations and a neutral, initially ionised molecule. In ETMD(2), the electron filling the vacancy and the electron being emitted originate from the same water molecule, leaving it in a doubly charged state. Note that while core-level ICD has been observed in various systems, to our knowledge no experimental signature of core-level ETMD has been reported yet. In addition to the direct decay of the inner-shell hole, ICD and ETMD can take place also from Auger final states if the internal excess energy permits [Fig. 1(e) and (f)].^12,13,32^ Here, instead of a core-level, a valence-level vacancy is filled. While the kinetic energy of electrons emitted in core-level ICD or ETMD are comparable to Auger electron energies, ICD or ETMD electrons from Auger final states have considerably less energy, typically between zero to a few tens of eV.^26,33^

While in the case of inner-shell ionisation of pyrimidine the intermolecular processes “protect” the molecule from fragmentation by energy and charge transfer to the water surrounding,^19^ it is now an intriguing question what happens if the X-ray photon targets the water environment instead. Will intermolecular processes in turn lead to ionisation and fragmentation of the pyrimidine? To address these questions, we core-ionised the water in a heterogeneous pyrimidine–water cluster, recorded the resulting photoelectron–photoion–photoion coincidence (PEPIPICO) spectra and combined the measurement with calculated electron spectra.

## Results and discussion

**Fig. 2:**
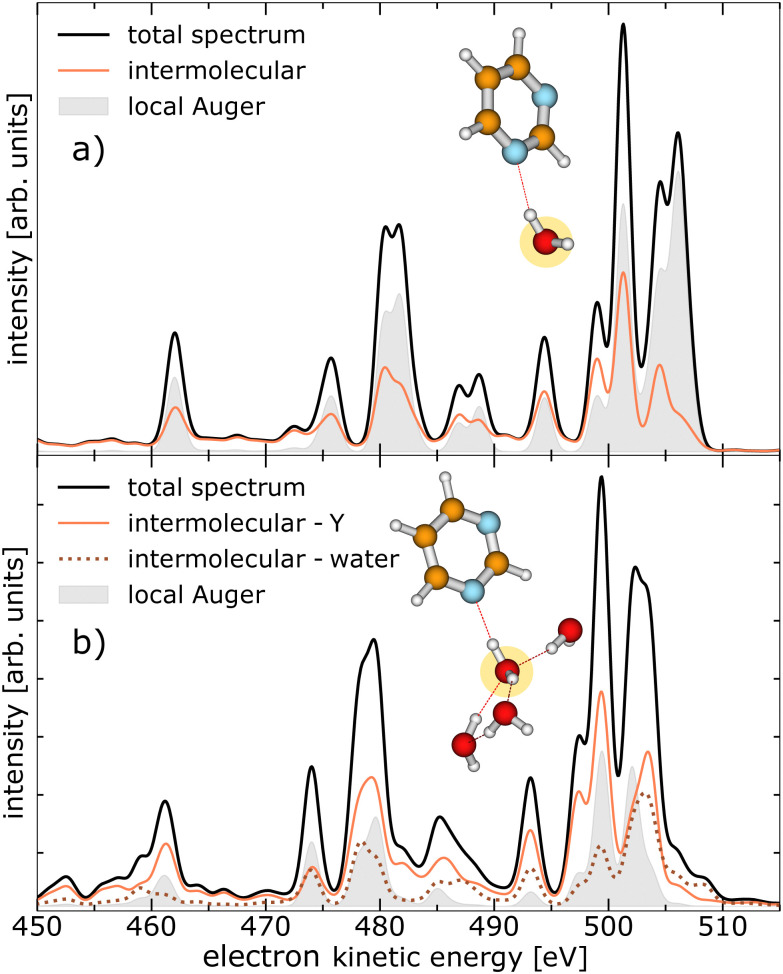
Theoretical calculation of the kinetic-energy spectrum of a pyrimidine–H_2_O dimer in panel (a) and a pyrimidine–(H_2_O)_4_ cluster in panel (b) after O 1s photoionisation, broken down by the nature of the final states. The calculated equilibrium geometry of the clusters is shown and the targeted O atom is marked yellow. The total electron emissions are shown (black trace) with the contributions of the local Auger decay (filled gray trace) and intermolecular processes involving the pyrimidine, abbreviated as Y (orange solid line). The intermolecular processes exclusively between water molecules are presented as brown dotted line in panel (b).

Similar to ref. 19 we performed calculations on the strengths of the different local and non-local decay channels contributing to the decay of the O 1s vacancy in pyrimidine–(H_2_O)_*n*_ complexes. The simulated electron emission spectra are displayed in Fig. 2, for one water molecule in the vicinity of the pyrimidine in panel (a), and for four water molecules in panel (b). The equilibrium geometries of the two pyrimidine–water complexes are illustrated in the insets, with the core-ionised O atom marked in yellow. The kinetic-energy spectrum of all expected electrons (black solid lines) is shown with the contributions of the local Auger decay (filled gray traces), the intermolecular processes involving pyrimidine (orange solid lines), and the intermolecular processes only including water molecules [brown dotted line in panel (b)]. As is evident from Fig. 2, the intermolecular processes exhibit already a considerable share with only one water present (a) and outpace the local processes clearly in the presence of four water molecules (b). In the ESI,† the calculated electron spectra for pyrimidine in the vicinity of one to four water molecules are shown in more detail, including a breakdown of the intermolecular processes in their contributions of core-level ICD, core-level ETMD(2), and core-level ETMD(3).

**Fig. 3:**
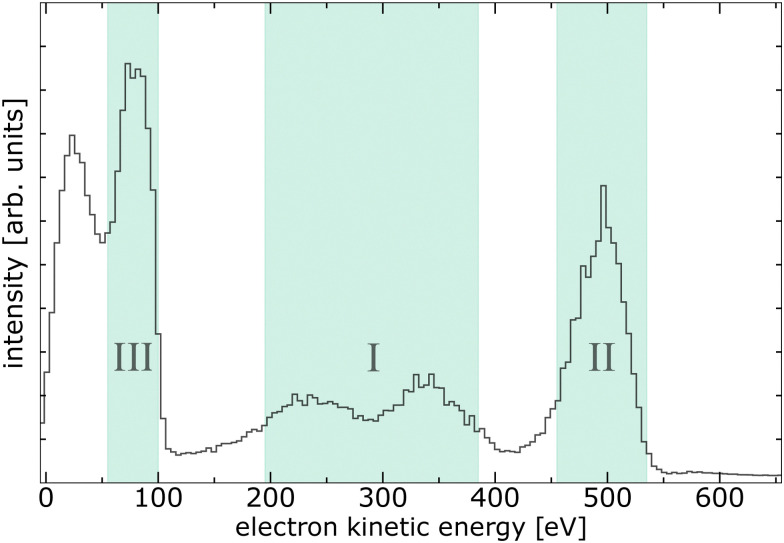
Electron spectrum from all detected PEPIPICO events of a mixed pyrimidine–water target at an exciting-photon energy of 620 eV. Three regions are indicated with electrons originating from different processes used as filter conditions for the ion–ion coincidence maps in Fig. 4(a)–(c).

Experimentally, we measured PEPIPICO spectra from pyrimidine–water clusters at an exciting-photon energy of 620 eV. The corresponding electron spectrum in coincidence with two ions of arbitrary mass-to-charge ratios (*m*/*q*) is shown in Fig. 3. The relatively poor resolution of the spectrum is a result of the applied extraction voltages required for the ion detection (see Experimental section and ref. 19 for details). Nevertheless, it is sufficient to distinguish certain features: the O 1s photoelectrons (region III in Fig. 3, 55–100 eV), electrons related to emission from pyrimidine, both inner-shell photoelectrons and secondary electrons (region I, 195–385 eV), and electrons ejected in the decay of water core-level vacancies (region II, 455–535 eV). In addition to these three main features, a faint signal close to 600 eV contains all photoemitted valence electrons. Finally, another peak around 20 eV can be identified, which is mainly formed by high-energy electrons (*e.g.*, photoelectrons or Auger electrons) that have lost a substantial amount of their energy through inelastic scattering, by electrons created from electron-impact ionisation, and by electrons emitted through valence-level ICD or ETMD. Note that other weak processes with non-discrete electron spectrum like double Auger decay may lie below the main features. For region III (55–100 eV) associated to the O 1s photoelectron peak, with a nominal kinetic energy at about 82 eV,^34–36^ the signal of slow electrons extends significantly into this region. In region I (195–385 eV), we expect photoelectrons and Auger electrons emitted after the C 1s [binding energies of 291.09 eV, 292.08 eV, and 292.48 eV (ref. 37)] and N 1s [binding energy of 405.23 eV (ref. 38)] ionisation of pyrimidine as well as electrons related to non-local processes initiated by these inner-shell ionisations.^19^ Electrons in region II (455–535 eV) result from Auger and non-local decays after core ionisation of water, either of free, gaseous water contained in the jet, or bound in homogeneous (H_2_O)_*n*_ or heterogeneous pyrimidine–(H_2_O)_*n*_ clusters.

**Fig. 4:**
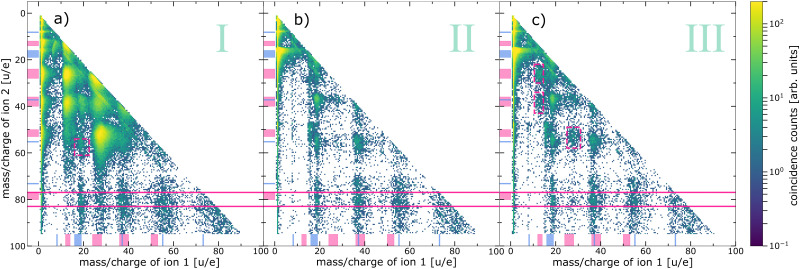
Ion–ion coincidence maps recorded at an exciting-photon energy of 620 eV coincidently measured with electrons of different kinetic-energy ranges. (a) Ion–ion pairs in coincidence with electrons resulting from pyrimidine core-level processes (region I in Fig. 3), (b) with water core-level processes (region II in Fig. 3, excluding the O 1s photoelectrons), and (c) with the O 1s photoelectrons (region III in Fig. 3). Note the logarithmic colour scale. The ranges of expected fragments originating from pyrimidine (pink) and water monomers or clusters (blue) according to Table 1 are indicated with respective markers at both axes. The horizontal pink solid lines indicate the region of coincidences of an intact pyrimidine parent ion (*m*/*q*_ion2_ = 80 ± 3 u/e) and a second ion. Some additional features are highlighted with pink dashed boxes, for details see text.

The *m*/*q* of the main fragment ions resulting from inner-shell ionisation of water or pyrimidine are listed in Table 1. As evident from the table, almost all expected fragments are singly charged. The probability to detect doubly or triply charged pyrimidine fragments is low due to the instability of these fragments. It is further evident that the expected features from some of the ion fragments overlap or are close in their *m*/*q* making some assignments challenging. This is not the case, however, for the pyrimidine parent ion with a *m*/*q* of 80 u/e. The corresponding maps of ion–ion pairs selected for coincidences with electrons of the three indicated regions are shown in Fig. 4(a)–(c).

**Table 1:** Mass-to-charge ratio (*m*/*q*) of the main fragment ions expected from inner-shell ionisation of a pyrimidine–water mixture. The *m*/*q* of the pyrimidine fragments was taken from ref. 22. Many of the pyrimidine fragments and water-cluster ions overlap in their *m*/*q*. The intact pyrimidine ion can be found at a *m*/*q* = 80 u/e

Mass-to-charge ratio [u/e]	Corresponding ions
1	H^+^
8	O^2+^
12–14	C^+^/CH^+^/N^+ ^(ref. 22)
16–19	O^+^/OH^+^/H_2_O^+^/H_2_OH^+^
24–28	C_2_^+^/C_2_H^+^/C_2_H_2_^+^/CN^+^/CHN^+^/CH_2_N^+ ^(ref. 22)
37	(H_2_O)_2_H^+^
36–40	C_3_^+^/C_3_H^+^/C_3_H_2_^+^/C_3_H_3_^+^/C_2_N^+^/C_2_HN^+^/C_2_H_2_N^+^/CN_2_^+ ^(ref. 22)
50–53	C_3_N^+^/C_3_HN^+^/C_3_H_2_N^+^/C_3_H_3_N^+^/C_2_N_2_^+^/C_2_HN_2_^+ ^(ref. 22)
55	(H_2_O)_3_H^+^
80	C_4_H_4_N_2_^+^ (pyrimidine parent ion)

Fig. 4(a) exhibits all events of two ions in coincidence with electrons of region I of Fig. 3, emitted after inner-shell ionisation of the C or N atoms of the pyrimidine. A similar ion–ion map can be found in ref. 19 recorded below the N 1s and O 1s edges. Both maps mainly show features originating from the fragmentation of pyrimidine into two cationic fragments (*m*/*q* = 12–14 u/e, 24–28 u/e, 36–40 u/e, and 50–53 u/e). These breakup channels agree well with previously reported fragment spectra of inner-shell-ionised gas-phase pyrimidine.^22,24,37^ Besides these main features, some additional observations are noteworthy: (i) one pyrimidine fragment in coincidence with a water-cluster ion (*m*/*q* = 16–19 u/e, 37 u/e, and 55 u/e), (ii) one intact pyrimidine molecule (*m*/*q* = 80 u/e) with water-cluster ions, and (iii) one intact pyrimidine molecule with pyrimidine fragments. These observations have been discussed previously and case (ii) was interpreted as a protective effect of the water surrounding the biomolecule due to the intermolecular core-level processes.^19^ Another weak but interesting feature is the coincident emission of an ion pair with ion 1: *m*/*q* = 16–19 u/e and ion 2: *m*/*q* = 55 u/e, both assigned to water-cluster ions and highlighted with a pink box in Fig. 4(a). This feature has not been discussed before and provides evidence for core-level ETMD(3): despite C 1s or N 1s ionisation, no pyrimidine fragment is observed, but two water-cluster ions are formed instead. This interpretation is, however, tentative, because the signal is weak and the resolution is poor. An alternative way to produce two water cations is valence ETMD(3) subsequent to Auger decay, if the pyrimidine parent ion is missed in detection.

The ion–ion map of Fig. 4(b) contains ion pairs in coincidence with an electron emitted in a water O 1s decay. In comparison to Fig. 4(a) the overall map differs significantly, mainly because of the absence of two pyrimidine fragment ions in coincidence. The main feature originates here from coincidences of two fragments of the water monomer. As expected, we also observe signal resulting from two water-cluster fragments. Interestingly, there is significant signal from an intact pyrimidine ion with water-cluster fragments (between pink solid lines). Therefore, we can conclude that an intermolecular energy or charge transfer has taken place initiated by an O 1s ionisation and ending with a singly ionised pyrimidine. Surprisingly, no clear signal of pyrimidine fragments in coincidence with any other ion can be found in this ion–ion map.

Fig. 4(c) depicts the ion–ion map in coincidence with the O 1s photoelectron. Intuitively, this map should contain the same features as the ion–ion map in coincidence with the water core-level decay processes, *i.e.*, Fig. 4(b). By comparing both maps, we can identify three features present in Fig. 4(c) that are absent in Fig. 4(b), highlighted by pink dashed boxes. All of them can be assigned to one or two pyrimidine fragments. Taking into account the electron spectrum shown in Fig. 3, these pyrimidine fragments may result from coincidences with slow electrons (originating from pyrimidine or water) instead of coincidences with the O 1s photoelectrons. Owing to experimental challenges, the resolution in both electron and ion spectra is relatively low. The different decay channels can thus not be compared quantitatively. However, the presence of pyrimidine parent ions but absence of pyrimidine fragment ions in Fig. 4(b) allows some further conclusions. First, ETMD(2) of water O 1s vacancies with pyrimidine seems to be unlikely, since it would lead to doubly charged dissociative pyrimidine. Second, the observation of intact pyrimidine parent ions but no fragments is an evidence for core-level ICD or core-level ETMD(3), or ICD/ETMD(3) from Auger final states. In all cases, it seems that predominantly the four outermost valence orbitals of pyrimidine are ionised. These valence-ionised states are known to be stable, while deeper valence ionisation is dissociative.^39^ For an improved disentanglement and unambiguous (quantitative) assignment of local or non-local processes, a higher resolution in the electron spectrum of the PEPIPICO is crucial. Especially in combination with theory, the investigation and understanding of these decays can enhance our knowledge of the mechanisms of radiation damage on a molecular level. The distinction of non-local mechanisms, taking place directly in the decay of the core-level vacancy, and those from excited valence states subsequent to Auger decay seems feasible with high-resolution electron spectra. In this regard, high-resolution studies tackling this topic would be worthwhile to conduct.

## Conclusion

Summarizing, we measured PEPIPICO of core-ionised pyrimidine–water clusters. Taking advantage of the coincidence technique, clusters with core holes on either the pyrimidine or the water molecule could be differentiated *via* electron spectroscopy, allowing for the analysis of the resulting ions generated during the decay process. A previously described energy transfer from ionised pyrimidine to neighbouring water *via* non-local core-hole decay could be confirmed. Additionally, we identified the opposite scenario. Theoretically and experimentally, we observe water core-level ionisation and subsequent immediate or cascade non-local decay involving neighbouring pyrimidine or other water molecules. Interestingly, the pyrimidine molecules seem to end solely in singly charged non-dissociative states, as the absences of pyrimidine fragments are suggesting. Additionally, we found first experimental indications for core-level ETMD after initial C 1s/N 1s ionisation of the pyrimidine molecule in a cluster.

## Experimental

The experiment was performed at the P04 soft X-ray beamline of PETRA III (DESY, Hamburg, Germany). A photoelectron–photoion–photoion coincidence (PEPIPICO) setup was used, following the scheme of ref. 40.

For the experiment, a gaseous cluster jet was crossed orthogonally (in the horizontal plane) with the synchrotron beam. Orthogonal to the interaction region (vertically) the electron and ion spectrometers were mounted, the former upwards and the latter downwards.

The electron spectrometer of the PEPIPICO setup consists of a magnetic-bottle time-of-flight electron spectrometer, equipped with a ring-shaped permanent magnet, an approximately 910 mm long drift tube, where several retardation voltages can be applied, and a microchannel-plate detector. Opposite to the electron drift tube, a 23 mm long ion time-of-flight spectrometer is mounted. The ring-shaped permanent magnet is part of the ion spectrometer, extracting the ions towards their drift tube. The synchrotron was operated in the 40-bunch mode with 192 ns time spacing between two consecutive light pulses.

The gaseous cluster jet was produced by the supersonic coexpansion of a vapor mixture through a conical 80 μm nozzle with an opening angle of 30°. The mixture consists of pyrimidine–water vapor resulting from evaporation of a pyrimidine–water solution, heated to 80 °C and containing 94% water and 6% pyrimidine. The cluster jet consists of water and pyrimidine monomers, pure water clusters, water–pyrimidine clusters, and in very small quantities pure pyrimidine clusters. The expansion chamber was separated from the interaction chamber through a skimmer with a 0.7 mm opening. For a detailed description of the experiment as well as data acquisition and treatment, see ref. 19.

From typical jet velocities, the number of interactions per time, and the repetition rate of the synchrotron it can be ensured that the sample is “fresh” for every interaction, *i.e.*, no molecule interacts more than once with the X-rays. Due to the orthogonality between the cluster jet and the ion spectrometer, only ions created through the interaction with synchrotron radiation are directed toward the ion drift tube by the applied extraction voltages. In contrast, neutral molecules and neutral fragments remain unaffected by the extraction voltages and, as a result, cannot be detected.

The calibration of the measured ion spectra from time of flight to *m*/*q* in the PEPIPICO analysis was done by applying filters to the electron spectrum and then assigning *m*/*q* values to well-known features in the mass spectra.

## Computational details

The spectra of the electrons emitted by the decay of the O 1s vacancy were computed following the well-established methodology, used also in our previous work, ref. 19. Here, we briefly outline the main ingredients. The equilibrium geometries of the studied pyrimidine–water complexes were obtained using the second-order Møller–Plesset (MP2) method with the cc-pVTZ basis sets. The dicationic states populated by the decay of core-ionised water molecules in the pyrimidine–water complex were obtained using the statistical method for computing Auger spectra proposed in ref. 41. The dicationic states of the system were computed with the help of the second-order algebraic diagrammatic construction scheme [ADC(2)] for the calculation of the poles and residues of the particle–particle propagator^42,43^ using the cc-pVDZ basis sets. Depending on the localization of the two final holes, each state in the dicationic spectrum was then decomposed into different contributions attributed to Auger (two holes on the initially ionised water molecule), ICD (one hole on the initially ionised water molecule and one hole on the pyrimidine or another water molecule), ETMD(2) (two holes on the pyrimidine or another water molecule), and ETMD(3) (two holes distributed on two molecules different from the initially ionised water). As the number of final dicationic states of the pyrimidine–water complex is enormous, computing the individual decay rates is out of reach. To estimate the corresponding decay rates, we thus used the contribution in each transition moment of the configuration with two holes in the oxygen atom bearing the initial vacancy. Finally, the kinetic energies of the electrons emitted in the O 1s decay were obtained by subtracting the populated dicationic states from the energy of the O 1s core-hole state. The latter was computed by the means of the ΔSCF method using the cc-pVDZ basis sets. To account for the vibrational broadening and the experimental resolution, the resulting spectra were convoluted with a Gaussian function with FWHM of 1.5 eV.

## Author contributions

A. H. and A. K. conceived the experiment. A. H., P. S., J. H. V., C. K.-W., S. D., G. H., and F. T. performed the beamtime. D. B. did the data analysis. D. B., A. H., A. E., A. I. K., and L. S. C. discussed the results. N. V. K., A. I. K., J. M., and L. S. C. provided the theoretical calculations. D. B. drafted the manuscript, which was discussed and finalized by all authors.

## Data availability

The data generated in this study have been deposited in a Zenodo database https://doi.org/10.5281/zenodo.14418487.

## Conflicts of interest

The authors declare no competing financial interest.

## Supplementary Material

CP-027-D4CP03907F-s001
