# Supplementary material for: Interplay of protection and damage through intermolecular processes in the decay of electronic core holes in microsolvated organic molecules
Source: Phys Chem Chem Phys. 2025 Mar 6;27(18):9329–35. doi: 10.1039/d4cp03907f (PMC11883753; doi:10.1039/d4cp03907f)
Supplement: CP-027-D4CP03907F-s001 [file CP-027-D4CP03907F-s001.pdf]

Supplementary Information (SI) for Physical Chemistry Chemical Physics.
This journal is © the Owner Societies 2025

Supplementary Information - Interplay of protection and damage through intermolecular processes in the decay of electronic core holes in microsolvated organic molecules

Dana Bloß*,[1] Nikolai V. Kryzhevoi,[2] Jonas Maurmann,[2] Philipp Schmidt,[3] André Knie,[1] Johannes H. Viehmann,[1] Catmarna Küstner-Wetekam,[1] Sascha Deinert,[4] Gregor Hartmann,[5] Florian Trinter,[6,7] Lorenz S. Cederbaum,[2] Arno Ehresmann,[1] Alexander I. Kuleff,[2] and Andreas Hans+[1]

[1]*Institute of Physics and Center for Interdisciplinary Nanostructure Science and Technology (CINSaT), University of Kassel, Heinrich-Plett-Straße 40, 34132 Kassel, Germany*

[2]*Theoretical Chemistry, Institute for Physical Chemistry, Heidelberg University, Im Neuenheimer Feld 229, 69120 Heidelberg, Germany*

[3]*European XFEL, Holzkoppel 4, 22869 Schenefeld, Germany*

[4]*Deutsches Elektronen-Synchrotron (DESY), Notkestraße 85, 22607 Hamburg, Germany*

[5]*Helmholtz-Zentrum Berlin (HZB), Albert-Einstein-Straße 15, 12489 Berlin, Germany*

[6]*Molecular Physics, Fritz-Haber-Institut der Max-Planck-Gesellschaft, Faradayweg 4-6, 14195 Berlin, Germany*

[7]*Institut für Kernphysik, Goethe-Universität Frankfurt, Max-von-Laue-Straße 1, 60438 Frankfurt am Main, Germany*

THEORETICAL ELECTRON SPECTRA

Figures S1, S2, S3, and S4 show the theoretical electron spectra after O 1s ionisation of a pyrimidine-water cluster with one to four water molecules in detail. The geometries of the clusters are shown as insets in the corresponding figures. In all figures, the black solid traces present the total electron spectra, the gray filled traces the Auger spectra, the pink dotted traces the core-level ICD spectra, the blue dashed traces the core-level ETMD(2) spectra, and the green dashed-dotted traces the core-level ETMD(3) spectra. For Figs. S2-S4, the spectra on the left side illustrate the intermolecular processes with holes at the pyrimidine in the final states and the spectra on the right side the intermolecular processes with holes only at water molecules in the final states. The different rows correspond to different ionised water molecules (if the cluster contains more than one water molecule) and the ionised water is indicated by a black arrow. For a detailed description of the theoretical methods see the Experimental section of the main article.

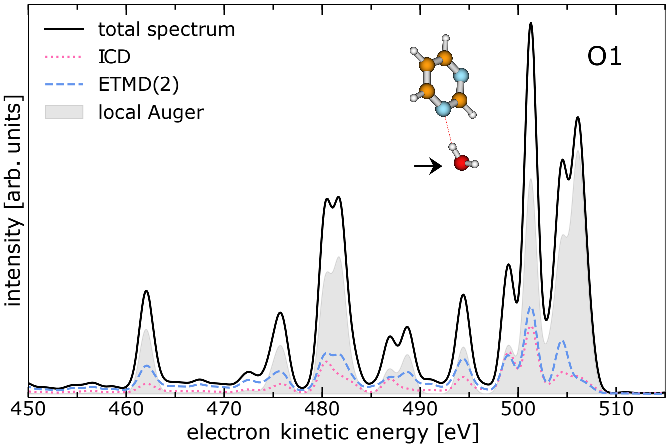

FIG. S1. Theoretical spectra of the emitted electrons in the decay of an O 1s vacancy in a pyrimidine-water dimer. The equilibrium geometry of this dimer is illustrated as an inset. The different local (Auger decay - gray filled trace) and intermolecular contributions (core-level ICD - pink dotted trace and core-level ETMD(2) - blue dashed trace) of the total spectra (black solid trace) are presented. In the dimer, all non-local decay paths necessarily imply holes at the pyrimidine in the final state.

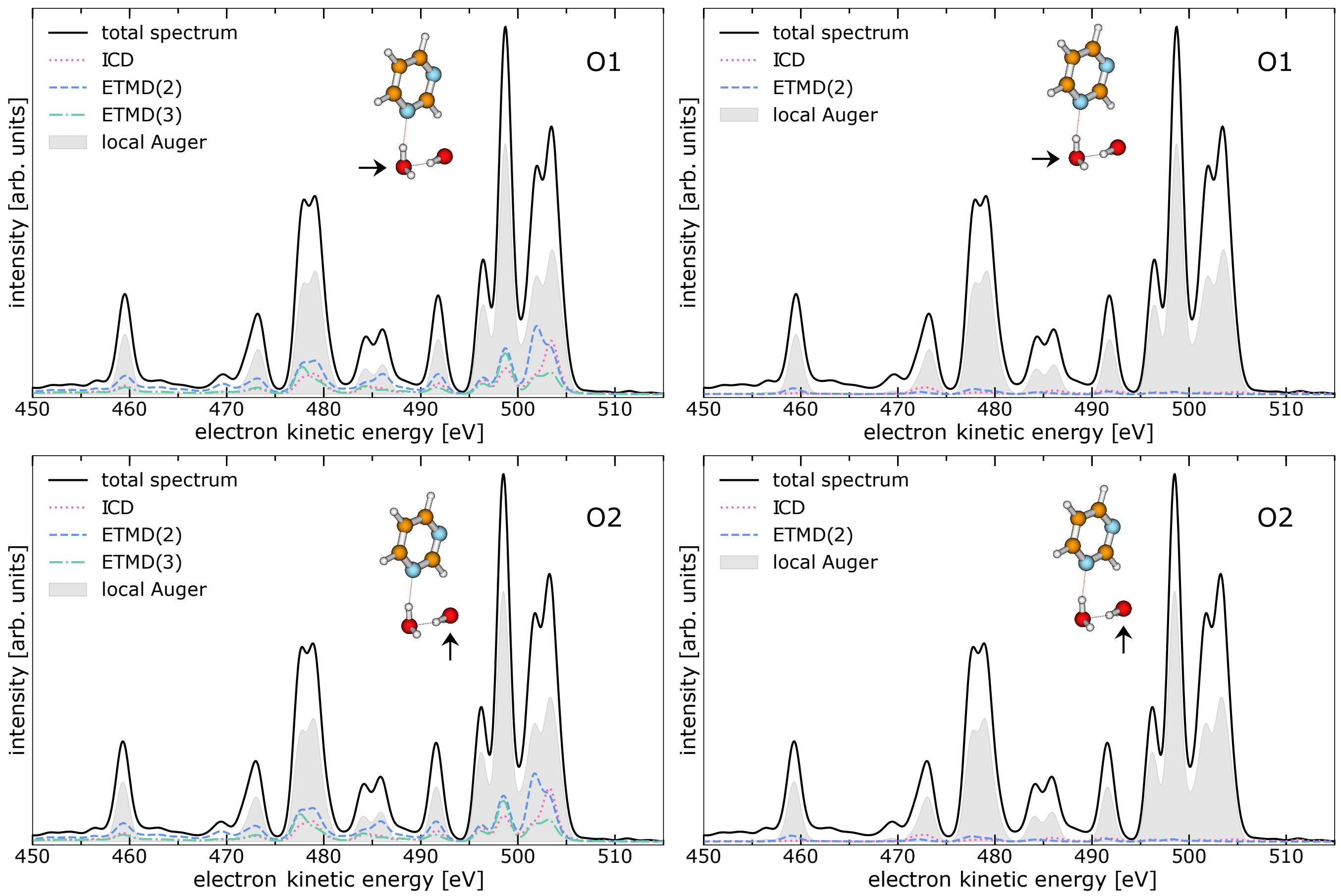

FIG. S2. Theoretical spectra of the emitted electrons in the decay of an O 1s vacancy in a pyrimidine-water cluster, consisting of one pyrimidine molecule and two water molecules. The equilibrium geometry of the cluster is illustrated as insets. The different local (Auger decay - gray filled traces) and intermolecular contributions (core-level ICD - pink dotted traces, core-level ETMD(2) - blue dashed traces, and core-level ETMD(3) - green dashed-dotted traces) of the total spectra (black solid traces) are presented. The spectra on the left side show the intermolecular processes with holes at the pyrimidine in the final states and the spectra on the right side the intermolecular processes with holes only at water molecules in the final states. Note that identical total and Auger spectra are shown in all figures for comparison. The different rows depict spectra resulting from the ionisation of different water molecules (marked with black arrows).

* dana-bloss@uni-kassel.de

+ hans@physik.uni-kassel.de

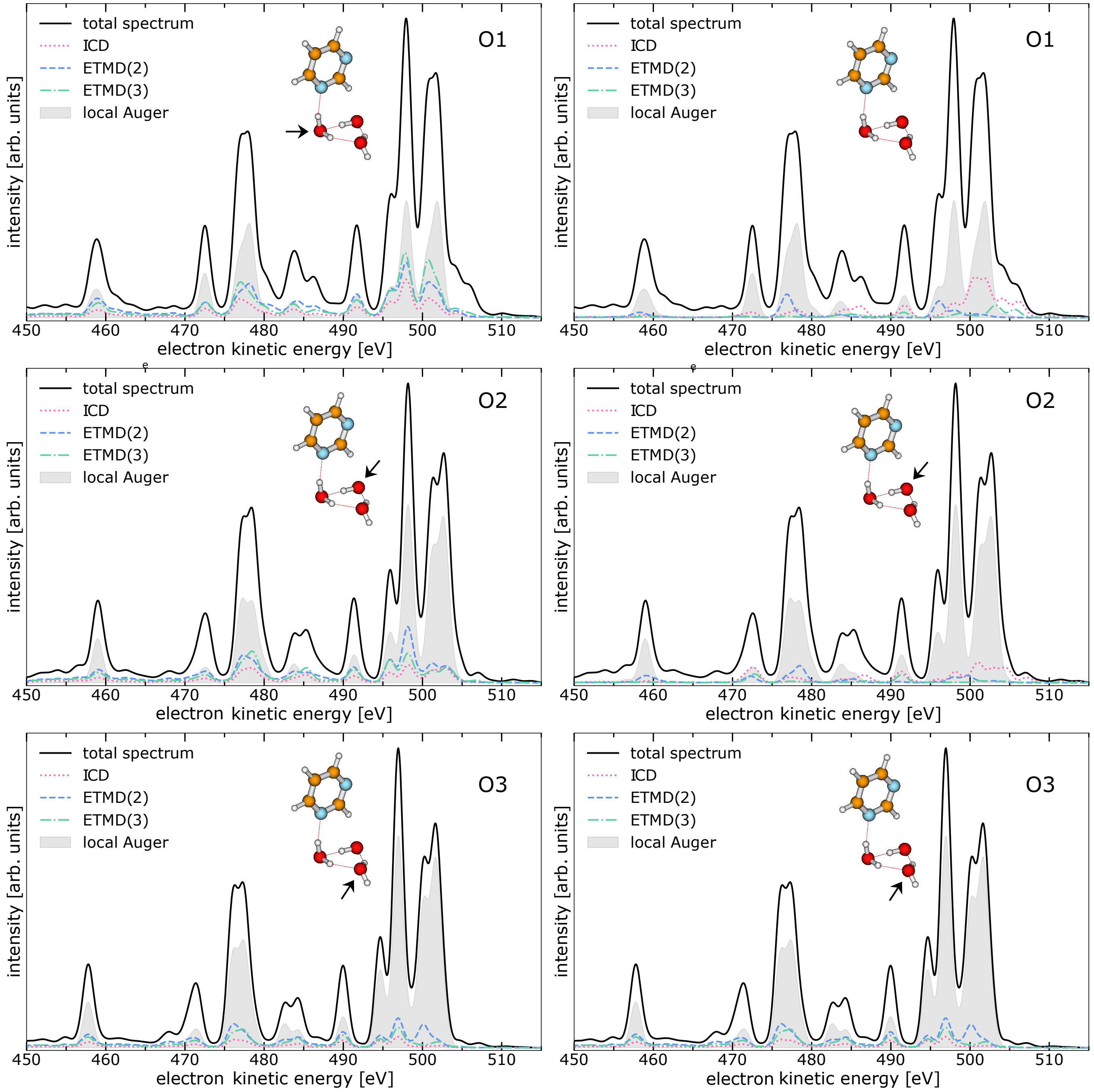

FIG. S3. Theoretical spectra of the emitted electrons in the decay of an O 1s vacancy in a pyrimidine-water cluster, consisting of one pyrimidine molecule and three water molecules. The equilibrium geometry of the cluster is illustrated as insets. The different local (Auger decay - gray filled traces) and intermolecular contributions (core-level ICD - pink dotted traces, core-level ETMD(2) - blue dashed traces, and core-level ETMD(3) - green dashed-dotted traces) of the total spectra (black solid traces) are presented. The spectra on the left side show the intermolecular processes with holes at the pyrimidine in the final states and the spectra on the right side the intermolecular processes with holes only at water molecules in the final states. Note that identical total and Auger spectra are shown in all figures for comparison. The different rows depict spectra resulting from the ionisation of different water molecules (marked with black arrows).

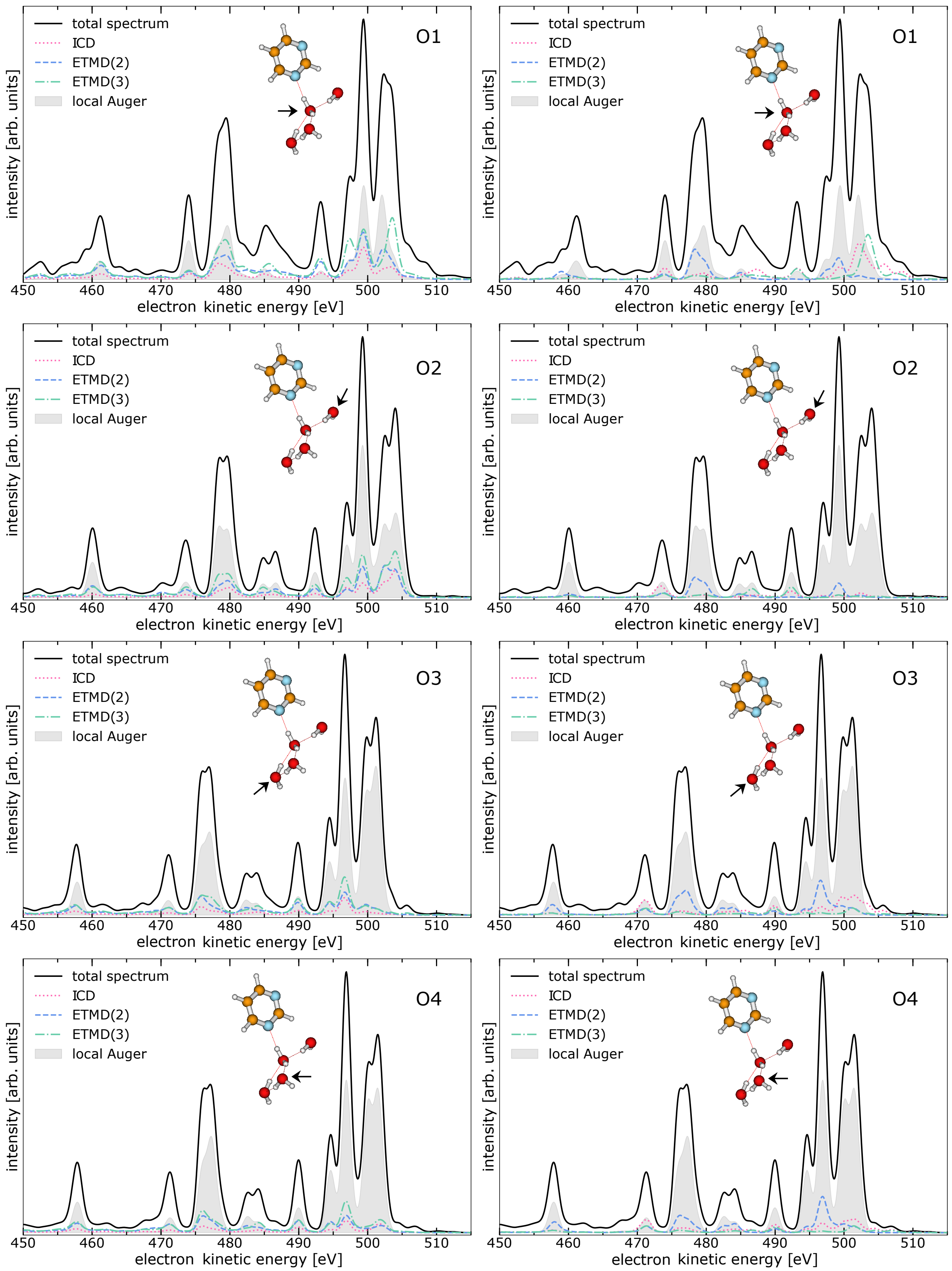

FIG. S4. Theoretical spectra of the emitted electrons in the decay of an O 1s vacancy in a pyrimidine-water cluster, consisting of one pyrimidine molecule and four water molecules. The equilibrium geometry of the cluster is illustrated as insets. The different local (Auger decay - gray filled traces) and intermolecular contributions (core-level ICD - pink dotted traces, core-level ETMD(2) - blue dashed traces, and core-level ETMD(3) - green dotted, dashed traces) of the total spectra (black solid traces) are presented. The spectra on the left side show the intermolecular processes with holes at the pyrimidine in the final states and the spectra on the right side the intermolecular processes with holes only at water molecules in the final states. Note that identical total and Auger spectra are shown in all figures for comparison. The different rows depict spectra resulting from the ionisation of different water molecules (marked with black arrows).
